# MRI Mouse Brain Data of Ischemic Lesion after Transient Middle Cerebral Artery Occlusion

**DOI:** 10.3389/fninf.2017.00051

**Published:** 2017-09-06

**Authors:** Inge A. Mulder, Artem Khmelinskii, Oleh Dzyubachyk, Sebastiaan de Jong, Marieke J. H. Wermer, Mathias Hoehn, Boudewijn P. F. Lelieveldt, Arn M. J. M. van den Maagdenberg

**Affiliations:** ^1^Department of Neurology, Leiden University Medical Center Leiden, Netherlands; ^2^Division of Image Processing (LKEB), Department of Radiology, Leiden University Medical Center Leiden, Netherlands; ^3^Percuros B.V., Department of Developmental Bio-Engineering, University of Twente Enschede, Netherlands; ^4^Department of Radiation Oncology, Netherlands Cancer Institute Amsterdam, Netherlands; ^5^Department of Human Genetics, Leiden University Medical Center Leiden, Netherlands; ^6^In-Vivo-NMR Laboratory, Max Planck Institute for Metabolism Research Cologne, Germany; ^7^Intelligent Systems Group, Faculty of Electrical Engineering, Mathematics and Computer Science, Delft University of Technology Delft, Netherlands

**Keywords:** ischemic stroke, MRI, mouse, data collection, brain

## Data—summary and highlights

In this data report we make available to the community a highly variable longitudinal MRI mouse brain data set of ischemic lesion after transient middle cerebral artery occlusion (*t*MCAo). Together with the provided semi-automated and automated segmentations, these data can be used to further improve the method proposed by Mulder et al. (2017) and also to serve as a benchmark for comparison between different approaches to segment ischemic lesions in MRI mouse brain data. It can also be used to develop and validate algorithms that further classify the stroke area into core and penumbra.

- The data were collected from mice: (i) of different ages, (ii) of two different strains, (iii) at different time points after the ischemic infarct induction, (iv) from two laboratories, (v) using two different MRI systems, and (vi) using three different sets of acquisition parameters.
- Segmentations of the ischemic lesions are provided as well. These were obtained by: (i) two observers using a semi-automated method and (ii) using the novel automated segmentation approach described by Mulder et al. (2017).
- Type/format of data: raw files, MetaImage files, text/Excel files, analyzed data.
- The following set of images associated with each of the 121 scans is included: raw Bruker MRI data (reference scan, T2 scan with all echoes, calculated T2-weighted map), automated segmentations of the ischemic lesions and semi-automated segmentations by two observers.
- For 99 of these scans, an accompanying set of Bruker MR diffusion maps, containing the Diffusion-Weighted Image (DWI) and calculated Apparent Diffusion Coefficient (ADC) maps, is included.
- Acquisition hardware: small-animal Bruker MRI systems (7 T and 11.7 T).
- Experimental set-up: infarct was induced in male mice of different age and background, using the *t*MCAo model. After that, MRI scans at different time points after infarct induction were acquired.
- Data sources: Leiden, Netherlands; Cologne, Germany.
- Data accessibility: all related data sets (121 T2 scans + template + 99 diffusion scans) were deposited in the public Dryad Digital Repository (https://doi.org/10.5061/dryad.1m528).

## Experimental design, materials and methods^1^

### Animals and MRI data

Male WT mice were subdivided into three main sets, respectively labeled as: “*Leiden-Set”*, “*Cologne-Set-1”*, “*Cologne-Set-2”*, depending on the city of origin and acquisition protocol.

*Leiden-Set:* C57BL/6J mice (*n* = 58) were further subdivided into three age groups (3- to 5-, 12- to 14- and 20- to 24-month-old). Mice were repeatedly scanned at different time points: 4 h, 24 h, 48 h and 8 d after infarct induction; see “Experimental Infarct Model” section.
*Cologne-Set-1:* C57BL/6J mice (*n* = 6; 3-month-old) were scanned at 18 h and 4 d after infarct induction.
*Cologne-Set-2:* Transgenic mice expressing luciferase under doublecortin control (DCX-Luc, Couillard-Després et al., 2008) (*n* = 10; 2- and 12-month-old) were scanned at 48 h after infarct induction.

Animals were housed with littermates, in a temperature-controlled environment, with food and water *ad libitum*. All animal experiments performed at the Leiden University Medical Center (LUMC) were approved by the local committee for animal health, ethics, and research of LUMC. All animal experiments conducted at the Max Plank Institute for Metabolism Research in Cologne were performed in accordance with the German Animal Welfare Act and approved by the local authorities (Landesamt für Naturschutz, Umwelt und Verbraucherschutz NRW).

Scans were performed with small-animal Bruker MRI systems using a Multi-Slice Multi-Echo sequence protocol. Animals from the *Leiden-Set* were scanned at 7 T (Pharmascan, Bruker BioSpin, Ettlingen, Germany), whilst animals from the *Cologne-Sets* were scanned at 11.7 T (Biospec 11.7 T/16, Bruker BioSpin). Quantitative T2 and ADC maps were calculated from the raw data using Paravision 5.1 software (Bruker Pharmascan) for the *Leiden-Set* and IDL software was used to calculate the quantitative T2 maps of the *Cologne-Sets*.

Table 1 shows a complete overview of all scans, together with a summary of the main imaging acquisition parameters.

**Table 1:** Summary of the data made available.

	**Data sets**															
	***Leiden-Set (LS)*** **C57BL/6J**										***Cologne-Set-1 (CS1)*** **C57BL/6J**			***Cologne-Set-2 (CS2)*** **DCX-Luc**		
Age (months)	3–5				12–14			20–24			3			2		12
Infarct induction	4 h	24 h	48 h	8 d	4 h	24 h	48 h	4 h	24 h	48 h	18 h		4 d		48 h	
Number: T2	11	36	8	6	7	9	8	5	7	5	6		3	5		5
Number: diffusion	11	35	7	6	7	9	7	5	7	5	–		–	–		–
**MRI ACQUISITION PARAMETERS**																
Magnet strength (T)	7						7				11.7			11.7		
Scan type	T2						DWI				T2			T2		
Repetition time (ms)	4,000						4,000				5,000			4,500		
Echo time (ms)	9						25				10.25			10		
Number of echoes	20						1				16			16		
Number of averages	2						1				1			1		
Field of view (mm^2^)	15 × 15						15 × 15				14 × 14			20 × 20		
Matrix	128 × 128						128 × 128				128 × 128			196 × 196		
Number of slices	16						16				10			10		12
Slice thickness (mm)	0.50						0.50				0.80			0.60		
Voxel size (mm^3^)	0.12 × 0.12 × 0.50						0.12 × 0.12 × 0.50				0.11 × 0.11 × 0.80			0.10 × 0.10 × 0.60		
Inter-slice gap (mm)	No gap						No gap				No gap			No gap		
Bandwidth (Hz)	59,523.8						50,000				50,000			75,000		
Acquisition time	12 min 48 s						17 min 4 s				10 min 40 s			9 min 18 s		
B value (s/mm^2^)	–						1,500				–			–		
**DIFFUSION-ENCODING GRADIENTS**																
Length δ (ms)	–						5				–			–		
Separation Δ (ms)	–						14				–			–		
Strength (mT/m)	–						267.34				–			–		
Direction	–						*x*-direction				–			–		

*Three sets of data were named depending on their origin and MRI acquisition protocol as: Leiden-Set, Cologne-Set-1 and Cologne-Set-2, respectively. Animals of different strains and ages were scanned at different time points after the infarct induction using different magnetic field strength and different MRI acquisition parameters. In total, 121 T2 scans (not counting the brain scan used as template) and 99 diffusion scans were made available*.

### Experimental infarct model

Infarcts were induced using a modified transient middle cerebral artery occlusion (*t*MCAo) model first described by Longa et al. (1989). Mice were anesthetized using isoflurane (3% induction, 1.5% maintenance) in 70% pressurized air and 30% O_2_. Painkiller carprofen (5 mg/kg, s.c.; Carporal, 50 mg/mL, AST Farma BV, Oudewater, Netherlands) was given before surgery. During surgery, the mouse body temperature was maintained at around 37°C using a rectal probe and feedback system. During the surgical procedure, a silicone-coated nylon monofilament (7017PK5Re; Doccol Company, Redlands, CA, USA) was inserted into the right common carotid artery and advanced via the internal carotid artery and circle of Willis to eventually block the middle cerebral artery (MCA) at its origin (decreasing blood flow substantially in the MCA territory, in the right hemisphere) and the skin was sutured. During the occlusion period, the mouse was allowed to wake up in a temperature-controlled incubator (V1200; Peco Services Ltd., Brough, UK). After 30 min of occlusion, the mouse was re-anesthetized in order to remove the suture and withdraw the monofilament to allow reperfusion. After surgery, the animal was allowed to recover for 2 h in the incubator to maintain body temperature at around 37°C, with easy access to food and water.

### Ischemic lesion segmentation

The novel method developed to segment the ischemic lesion in mouse brains in an automated fashion is described in detail in Mulder et al. (2017) and is available for download from the Software Downloads section of our webpage^2^. The same manuscript describes how the semi-automated segmentations (by two observers) used for validation of the automated algorithm were obtained. In “Description of the Files Associated with the Template Image” section, the files associated with the template brain image used as a part of the automated segmentation framework are described.

### Description of all scan names and corresponding MRI Bruker folders

All scans are named according to the following convention: “[*SetName*]_[*SubjectNumber*]_[*TimeAfterStrokeOnset*]”.

Examples of scan names (SN):

– LS_m53_24h
– CS1_m8_19h
– CS2_m4_2d

Bruker 2dseq data-files can be loaded using ImageJ^3^ with the Paravision 5.1 Bruker plug-in^4^ installed. In case of the *Leiden-Set*, the reference scan is located in the “\1\pdata\1” folder, the echoes and the calculated quantitative T2 maps are located in the “\2\pdata\1” and the “\2\pdata\2” folders, respectively. In case of the *Cologne-Set-1* and *Cologne-Set-2*, the echoes and T2 maps are located in the “\1\pdata\1” and the “\1\pdata\2” folders, respectively.

In case of the diffusion scans in the *Leiden-Set*, the reference scan is the same as the T2 reference scan and is located in the “\1\pdata\1” folder, the DWI maps (per effective *B*-value) are located in the “\3\pdata\1” folder and the calculated quantitative ADC maps: signal intensity (SI), standard deviation of SI, diffusion constant (mm^2^/seq), standard deviation of diffusion constant, standard deviation of the fitting curve, are located in the “\3\pdata\2” folder.

Inside the Bruker folders, all scans of each set were subdivided according to the different age groups.

### Description of the file names that correspond to the different types of data made available for each subject/scan

In addition to the Bruker MRI data files, corresponding echoes, T2 maps, automated ischemic lesion segmentations and the semi-automated ones obtained by two observers (IM, SdJ) are provided in MetaImage format. In addition, the semi-automated segmentations are also provided in a format readable by the ROI manager of ImageJ. Each file was named according to the following convention (here “SN” = “ScanName”): “[*SN*]_[*TypeOfDataDescriptor*]”:

– Echoes (NR = 1…20 for *Leiden-Set* or NR = 1…16 for *Cologne-Sets*): SN_echoe_NR(.mhd/.raw)
– T2 maps: SN_T2map(.mhd/.raw)
– Automated segmentations: SN_AUTOMATED(.mhd/.raw)
– Semi-automated segmentations:
  ◦ ImageJ: SN_RoiSet_IAM.zip, SN_RoiSet_SdJ.zip
  ◦ MetaImage: SN_MANUAL_IAM(.mhd/.raw), SN_MANUAL_SdJ(.mhd/.raw)

### Description of the files associated with the template image

All files associated with a single scan of the mouse, 24 h after stroke onset (belonging to the 3- to 5-month-old mice age group) that was used as a template/reference image in the registration step of the automated ischemic lesion approach proposed by Mulder et al. (2017), are provided in MetaImage format. The manually drawn labels were propagated to each data set and used to initialize the level-set-based segmentation. The sum of all the 20 echoes and the whole brain mask were used during the registration step as the fixed image and fixed mask, respectively:

– Sum-of-the-20-echoes image: Template_24h_SUM20echoes(.mhd/.raw)
– Whole brain mask: Template_24h_WholeBrainMask(.mhd/.raw)
– Echoes (NR = 1…20): Template_24h_echoe_NR(.mhd/.raw)
– T2 map: Template_24h_T2map(.mhd/.raw)
– Whole brain label: Template_24h_Label_1(.mhd/.raw)
– Ipsilateral hemisphere label: Template_24h_Label_2(.mhd/.raw)
– Ventricles label: Template_24h_Label_3(.mhd/.raw)
– Periventricular zone label: Template_24h_Label_4(.mhd/.raw)

## Author contributions

Conceived and designed the study: IM, AK, OD, MH, BL and AvdM. Performed animal experiments, *t*MCAo surgery and acquired MRI data: IM. Performed semi-automated ischemic lesion segmentation: IM and SdJ. Developed the algorithm for automated ischemic lesion segmentation in MRI mouse brain data after *t*MCAo occlusion and performed the automated segmentation: AK and OD. Analyzed segmentations and wrote the manuscript: IM, AK and OD. Commented on the manuscript: IM, AK, OD, MW, MH, BL and AvdM.

### Conflict of interest statement

The authors declare that the research was conducted in the absence of any commercial or financial relationships that could be construed as a potential conflict of interest.

## Footnotes

^1^This section was partially adapted from Mulder et al. (2017).

^2^http://www.lkeb.nl

^3^http://imagej.nih.gov/ij

^4^http://rsb.info.nih.gov/ij/plugins/bruker.html
